# A transcriptomic analysis of the effects of macrophage polarization and endotoxin tolerance on the response to *Salmonella*

**DOI:** 10.1371/journal.pone.0276010

**Published:** 2022-10-14

**Authors:** Katharine Sedivy-Haley, Travis Blimkie, Reza Falsafi, Amy Huei-Yi Lee, Robert E. W. Hancock

**Affiliations:** 1 Department of Microbiology and Immunology, University of British Columbia, Vancouver, British Columbia, Canada; 2 Department of Molecular Biology and Biochemistry, Simon Fraser University, Burnaby, British Columbia, Canada; Ohio State University, UNITED STATES

## Abstract

*Salmonella* is an intracellular pathogen causing significant morbidity and mortality. Its ability to grow inside macrophages is important to virulence, and is dependent on the activation state of the macrophages. Classically activated M1 macrophages are non-permissive for *Salmonella* growth, while alternatively activated M2 macrophages are permissive for *Salmonella* growth. Here we showed that endotoxin-primed macrophages (M^EP^), such as those associated with sepsis, showed similar levels of *Salmonella* resistance to M1 macrophages after 2 hr of intracellular infection, but at the 4 hr and 24 hr time points were susceptible like M2 macrophages. To understand this mechanistically, transcriptomic sequencing, RNA-Seq, was performed. This showed that M1 and M^EP^ macrophages that had not been exposed to *Salmonella*, demonstrated a process termed here as primed activation, in expressing relatively higher levels of particular anti-infective genes and pathways, including the JAK-STAT (Janus kinase-signal transducer and activator of transcription) pathway. In contrast, in M2 macrophages these genes and pathways were largely expressed only in response to infection. Conversely, in response to infection, M1 macrophages, but not M^EP^ macrophages, modulated additional genes known to be associated with susceptibility to *Salmonella* infection, possibly contributing to the differences in resistance at later time points. Application of the JAK inhibitor Ruxolitinib before infection reduced resistance in M1 macrophages, supporting the importance of early JAK-STAT signalling in M1 resistance to *Salmonella*.

## Introduction

*Salmonella enterica* is a Gram-negative enteric pathogen that can cause symptoms ranging from localized gastroenteritis to systemic infection and sepsis [1]. There are 95.1 million cases of non-typhoidal gastroenteritis globally [2]. Since *Salmonella* is primarily a foodborne pathogen, agricultural antibiotic use has increased the prevalence of antibiotic resistant *Salmonella* isolates in the food supply [3]. These resistant isolates are particularly dangerous when they cause septicemia or sepsis, with an estimated 535,000 cases of invasive (systemic) non-typhoidal *Salmonella* infection, causing 77,500 deaths in 2017 [2].

*Salmonella* is an intracellular pathogen, capable of producing a variety of effectors that suppress immune defenses and manipulate the host immune system to its benefit [1, 4, 5]. *Salmonella* effectors influence the host cytoskeleton, resulting in the internalization of the bacteria and the formation of a modified phagosome called the *Salmonella*-containing vacuole (SCV) [6]. Effectors associated with *Salmonella* Pathogenicity Island (SPI)-2 prevent the SCV from fusing with lysosomes [7], which are vesicular organelles containing the bactericidal phagocytic NADPH oxidase complex [6], thus protecting *Salmonella* from killing. Residence in the SCV prevents *Salmonella* from being detected by cytosolic receptors such as NLRP3 and NLRC4 inflammasomes, which would otherwise cause a pyroptotic response that can mediate host defense against *Salmonella* [8]. The ability of SCV-resident *Salmonella* to escape phagocytic defenses is important to the development of disease. *Salmonella* cannot progress to systemic infection without intracellular replication in phagocytes [9] that carry the bacteria to tissues such as the mesenteric lymph nodes, spleen, and liver [1]. In particular, virulence depends on *Salmonella*’s ability to survive inside macrophages [10].

*Salmonella*’s ability to survive and replicate within macrophages in turn depends on the macrophage polarization state [11, 12]. Macrophage activation occurs over a spectrum, with polarization states ranging from M1 to M2 [13, 14]. M1 macrophages, also termed classically-activated macrophages, are induced by bacterial lipopolysaccharide (LPS) and Th1 cytokines such as IFNγ, and have inflammatory and microbicidal functions. M2, or alternatively activated, macrophages are induced by Th2 cytokines such as IL-4 and Il-13, as well as IL-10, and have wound healing and anti-inflammatory functions. Macrophages with M1-like and M2-like profiles have been observed *in vivo* [14]. For example, during intestinal inflammation, macrophages with M1-like pro-inflammatory activity are temporarily recruited [15]. M2 macrophages can allow intracellular infection [13, 16] and are permissive to replication of intracellular *Salmonella*, while M1 macrophages are non-permissive for such growth [11, 12].

Sepsis, caused by a dysfunctional response to infection (including *Salmonella* infection), is characterized by an additional form of alternatively activated, or M2-like, macrophage: the endotoxin tolerant macrophage [17]. Following an initial exposure to LPS or analogous bacterial signature molecules, primed macrophages (termed here M^EP^), upon a second similar stimulus, display cellular reprogramming, which leads to reduced responsiveness to subsequent stimulation [18]. The contribution of endotoxin tolerance to the pathology of sepsis is likely complex. Desensitization to subsequent stimuli may initially protect against tissue injury and mortality caused by hyperinflammation [18] such as that observed in the early phases of sepsis [19], while promoting healing [20], suggesting a protective role for endotoxin tolerance. However, in the later phases of sepsis, mortality is typically associated with immune suppression [19], and endotoxin tolerance is associated with reduced *in vitro* killing of e.g. intracellular pathogen *Leishmania major* [21]. At the same time, endotoxin tolerance may preserve some antimicrobial functions, since mice stimulated with LPS prior to infection showed improved clearance of both *Pseudomonas aeruginosa* [22] and *S*. *enterica* [23]. Based on these findings, it has been uncertain as to whether endotoxin tolerant macrophages would be expected to be resistant or permissive for intracellular *Salmonella* replication.

Immunomodulatory treatments might be useful in treating the immune suppression associated with sepsis [19], and could represent an alternative to antibiotics when treating drug-resistant *Salmonella* infection. However, immunomodulatory therapies, including previous attempts at sepsis therapies, have been limited to date by our incomplete understanding of the highly complex systems regulating innate immunity and inflammation. *Salmonella* contains multiple pathogen signatures and bacterial effectors, including LPS, flagellin, lipoproteins, CpG-DNA, and the SPI-1 needle complex rod protein, which activate at least nine different pattern recognition receptors [24]. The immune response is further modulated by host molecules including cytokines such as IFNγ, and danger molecules such as heat shock proteins; these pathways sometimes overlap with those activated by bacterial molecules [25]. The mechanisms by which the integration of these different signals occur are not well understood, and some signaling molecules appear to have different roles depending on the extracellular environment and activity of other signals [26]. Moreover, the involved pathways contain feedback and feed-forward regulatory mechanisms and there is crosstalk between pathways. Overall, more than 2,000 genes have been identified as dysregulated in response to immune stimuli [27].

To enable treatment of *Salmonella* infection and *Salmonella* septicemia, it is necessary to develop a systems-level understanding of the responses to *Salmonella* of differently polarized macrophages. Here we determined *Salmonella* resistance in human monocyte-derived macrophages and applied transcriptomics to provide a systems-level analysis of the gene expression of differently polarized macrophages in response to *Salmonella* infection. This enabled the identification of mechanisms that might explain the observed differences in *Salmonella* resistance between differently polarized macrophages.

## Materials and methods

### Monocyte-derived macrophages (MDM) isolation/maturation

Blood samples were obtained from healthy donors who had provided written informed consent, under UBC Clinical Research Ethics Board approval, ethics certificate H04-70232. Peripheral blood mononuclear cells (PBMCs) were isolated from human blood using Lymphoprep (StemCell Vancouver, Canada) density gradient isolation. PBMCs were plated into 24-well plates at 2 x 10^6^ cells/mL in serum-free RPMI media supplemented with 2mM L-Glutamine and 1mM sodium pyruvate. Cells were incubated at 37°C, 5% CO_2_ for 1 hr, after which media was replaced with RPMI containing 10% heat-inactivated FBS (Gibco Waltham, USA) plus 2mM L-glutamine and 1mM sodium pyruvate (cRPMI), and 10ng/mL M-CSF (R&D Minneapolis, USA). The resulting monocytes were matured for 7 days in this media, changing media on days 3 and 5. This resulted in MDMs at a final density of 0.42–2.6 x 10^5^ cells/well, varying by donor.

MDM were polarized on day 7 by removing the media, washing vigorously in PBS, and replacing with cRPMI containing 50ng/mL IFNγ (Biolegend San Diego, USA) for M1, 50ng/mL IL-4 (Biolegend San Diego, USA) for M2, or 10ng/mL lipopolysaccharide (LPS) for M^EP^ macrophages. The LPS used in this experiment was extracted from *Pseudomonas aeruginosa* PAO1 strain H103, using the Darveau-Hancock method [28]. Samples were then incubated for 24 hr. For JAK-STAT inhibition, Ruxolitinib 10μM (MedChemExpress Monmouth Junction, USA) or 0.1% DMSO vehicle control were added along with the polarizing treatments. Supernatants were collected after 24 hr for measurement of cytokines using ELISA.

### Bacterial culture

*S*. *enterica* serovar Typhimurium strain SL1344 carrying plasmid (*ssaG*::GFP), was received from Dr. Christine Hale, Wellcome Trust Sanger Institute, and used to confirm SCV formation [29]. This strain was cultured in Luria-Bertani (LB) medium, or on LB-agar plates, supplemented with ampicillin 100μg/mL. For other experiments a constitutively GFP-expressing *S*. Typhimurium strain SL1344 mutant designated MCS003 (SL1344{Tn7 PA1lacO:sgfp2 t0 t1 FRT-cat-FRT t0 t1}), was obtained from Andrew Santos, UBC. MCS003 was cultured in LB supplemented with streptomycin 100 μg/mL and chloramphenicol 30 μg/mL. Cultures of these bacteria were grown overnight under static conditions at 37°C.

### Infection and sample collection

Intraceullar survival was assessed using a gentamicin protection assay, where in addition to collection of lysates for CFU counting, supernatants were collected for ELISA and lysates were collected for RNA isolation. Polarized MDM were washed once with sterile PBS, and media was replaced with 900 μL cRPMI. Plates were returned to the incubator (37°C, 5% CO_2_) for less than 1 hr while the bacterial culture was prepared for infection. *Salmonella* concentration was measured by taking the OD_600_, and bacteria were diluted to a calculated multiplicity of infection (MOI) of 20:1 per 100μL of bacterial dilution. Next-day colony counts indicated the actual multiplicity of infection averaged 27:1. For the uninfected condition, 100μL cRPMI media was instead added to wells. Following *Salmonella* addition, plates were swirled and centrifuged at 1,500 rpm for 5 min to distribute and sediment bacteria. Plates were returned to the incubator for 30 min to allow association of the bacteria with MDM. Samples were collected immediately thereafter (0 hr), or the medium was removed and replaced with cRPMI + 50μg/mL gentamicin. Intracellular infection progressed a further 2, 4, or 24 hr. For 24 hr samples, medium was removed at 2 hr and replaced with medium containing 10μg/mL gentamicin as described previously [12], for the remaining 22 hrs of incubation.

After incubation, supernatants were collected for ELISA. For bacterial quantification, macrophage cells were washed twice with PBS, and lysed using 1mL 0.1% Triton-X-100. Lysates were subjected to four 1:10 serial dilutions in sterile PBS, and 3–4 spots of 10 μL volume were applied to LB plates containing the appropriate selective antibiotics as outlined above. Plates were incubated at 37°C overnight, and colonies were counted.

For RNA isolation, macrophage cells were immediately treated with 300μL RNAProtect (Qiagen Germantown, USA), collected into sterile RNAse-free tubes, and pelleted by centrifugation at 400 x *g* for 5 minutes using a MicroCL 21R microcentrifuge. Macrophages were lysed using 350μL of lysis buffer from an RNAeasy Plus Mini kit (Qiagen Germantown, USA), with added β-mercaptoethanol, and lysate was stored at -80°C.

### ELISA

Supernatants were frozen at -20°C prior to measurement of cytokines and chemokines using ELISA. Primary and secondary (biotinylated) antibodies and standards were from eBioscience through Fisher (Waltham, USA), except for the MCP-1 standard which was from R&D as noted: TNFα (Fisher primary antibody cat. no. 14-7348-85, secondary antibody cat. no. 13-7349-85, cytokine standard solution cat. no. 14-8329-63), IL-6 (Fisher primary antibody cat. no. 14-7069-81, secondary antibody cat. no. 13-7068-85, cytokine standard solution cat. no. 14-8069-62), IL-10 (Fisher primary antibody cat. no. 14-7108-81, secondary antibody cat. no. 13-7109-81, cytokine standard solution cat. no. 14-8109-62), MCP-1 (Fisher primary antibody cat. no. 14-7099-85, secondary antibody cat. no. 13-7096-85, cytokine standard solution R&D cat. no. 279-MC), and IL-1β (Fisher primary antibody cat. no. 14-7018-85, secondary antibody cat. no. 13-7016-85, cytokine standard solution cat. no. 29-8108-60). Manufacturer’s protocols were followed for ELISAs, with optimization of antibody concentrations, sample dilutions, and incubation times performed in the laboratory. Capture antibodies were diluted 1:1000 (IL-6 and IL-10), 1:500 (IL-1β and MCP-1), or 1:250 (TNFα); detection antibodies were diluted 1:1000 (MCP-1), 1:500 (TNFα, IL-6, and IL-10), or 1:250 (IL-1β). Plates were incubated with samples for 1 hr, except for MCP-1 which incubated for 2 hr. Absorbance was read on an Epoch plate reader and fitted to a 4-parameter non-linear standard curve using Gen5 software (version 2.07 and 3.05).

### RNA isolation and RNA-Seq

RNA-Seq followed the standard operating procedures established in our laboratory [30]. Briefly, RNA was isolated from MDM lysates stored at -80°C using an RNAeasy Plus Mini kit (Qiagen, Germantown, USA) with DNAse treatment (RNAfree DNAse kit, Qiagen, Germantown, USA). RNA quality met sequencing standards upon analysis on an RNA 6000 Nano Chip on an Agilent 2100 Bioanalyzer (Agilent Technologies, Santa Clara, California, USA). From total RNA, mRNA was isolated using polyA enrichment with d(T) beads (New England Biolabs, Ipswich, Massachusetts, USA), and strand-specific cDNA libraries were created using a KAPA RNA HyperPrep kit (cat. no.: 07277253001, Roche, Basel, Switzerland). Library quality was assessed with an Agilent 2100 Bioanalyzer using a High Sensitivity DNA chip (Agilent, Santa Clara, California, USA) and libraries were sequenced on an Illumina HiSeq2500. Sequencing quality was assessed using *FastQC* (version 0.11.7) and *MultiQC* (version 1.0.dev0) [31], and sequence reads were aligned to the Ensembl human reference genome GRCh38 v91 [32] using *STAR* aligner (version 2.5.4b) [33], followed by read count generation using *HTSeq* (version 0.8.0) [34]. Four samples were removed due to having fewer than 1.2 million aligned reads, leaving n = 3 or 4 samples for each condition.

### Statistical analysis

Data were analyzed using R (version 3.6.0) [35] in RStudio, with visualization done using *tidyverse* (version 1.2.1) (https://cran.r-project.org/package=tidyverse) [36]. For CFU and ELISA data, values for each donor were indexed to the indicated reference samples to more accurately assess trends without interference from donor variability. Statistical significance was determined using the Mann-Whitney-Wilcoxon test.

RNA-Seq read counts were normalized, and Principal Component Analysis was used to verify that samples were separated by infection and polarization state as expected (S1 Fig). Differentially expressed genes were determined using *DESeq2* (version 1.24.0) [37] using paired analysis with the Wald test. Low read count genes, defined as those for which fewer than 3 samples contain 10 or more counts, were eliminated. Differentially expressed genes were considered to be those with a log_2_ fold change >1, with an adjusted p-value of <0.05 (with multiple test correction done using Benjamini-Hochberg). Ensembl gene identifiers and corresponding gene symbols were mapped using *org*.*Hs*.*eg*.*db* (version 3.8.2).

Gene-set analysis was performed using *roast*, employing 99,999 rotations [38], on counts normalized using *voom* [39] (*limma* version 3.40.2). Gene-sets used are described in S1 Table. Pathway analysis was performed using *Sigora* (version 3.0.1) [40] with pathway data from the *Reactome* repository [41] performing multiple test correction using the Bonferroni method. Networks based on protein:protein interactions were computed using *NetworkAnalyst* [42] with the *IMEx* Interactome [43], and *KEGG* [44], and visualized using *Cytoscape* [45].

## Results and discussion

### M1, M2, and M^EP^ macrophages expressed the expected markers, and M^EP^ macrophages upregulated endotoxin tolerance signature genes

Macrophage polarization into the M1 (inflammatory), M2 (wound healing), and M^EP^ (primed for endotoxin tolerance/reprogramming) types, was verified using cytokine production and expression of signature gene-sets. Over 24 hr of infection with *Salmonella*, M1 macrophages showed increased expression of the pro-inflammatory cytokines TNFα and IL-1β, as well as IL-6, when compared to M2 macrophages (S2 Fig), consistent with the expected pro-inflammatory nature of M1 macrophages [13, 14]. In contrast, M2 macrophages expressed higher levels of anti-inflammatory IL-10 than did M1 macrophages at both 4 hr and 24 hr after infection (S2 and S3 Figs), which was consistent with M2 polarization. A reduction of TNFα was observed in M^EP^ macrophages compared to both M1 and M2 macrophages at 4 hr after infection (S3 Fig), while at 24 hr low expression of both TNFα and IL1-β was observed; reduced expression of these cytokines is characteristic of endotoxin tolerance [20]. Thus cytokine expression indicated a correct polarization of these macrophages.

The endotoxin tolerance signature was identified in our lab [17] as a differentially expressed gene-set that occurred in endotoxin tolerant macrophages (treated twice with LPS, for 24 and 4 hr), but not in inflammatory (M1) macrophages (treated once with LPS, for 4 hr). It was found to be predictive of human sepsis and multi-organ failure at first clinical presentation in the emergency ward, in both a 500-patient retrospective analysis and a small (72 patient) prospective clinical study [17]. Gene-set testing with *roast* showed that endotoxin primed M^EP^ macrophages upregulated genes from this endotoxin tolerance signature when compared to M1 and M2 macrophages, both in infected and uninfected cells (Table 1). No other comparison showed an upregulation in these genes.

**Table 1:** *Roast* gene-set enrichment test for the endotoxin tolerance signature.

Macrophage Types Compared^1^	% Up-regulated^2^	% Down-regulated^2^	Direction of expression^3^	p-value^4^
M1 vs. M2 uninfected	31	16	Up	0.16
**M**^**EP**^ **vs. M1 uninfected**	**48**	**13**	**Up**	**<0.001**
**M**^**EP**^ **vs. M2 uninfected**	**53**	**13**	**Up**	**<0.001**
M1 vs. M2 infected	21	22	Down	0.83
**M**^**EP**^ **vs. M1 infected**	**49**	**11**	**Up**	**<0.001**
**M**^**EP**^ **vs. M2 infected**	**49**	**16**	**Up**	**<0.001**

^1^ Cell types and conditions for which gene expression counts were compared to determine enrichment.

^2^ Percentage of the genes in the signature that were found to be up- or down-regulated for the comparison in question by *roast*.

^3^ Whether the signature was found to be overall up-or down-regulated.

^4^ p-value was calculated by *roast* taking into account both percentage of up- and down-regulated genes and magnitude of change. Since 99999 rotations were performed the lowest possible p value was 0.001; Statistically significant (p<0.05) comparisons are bolded.

The statistically significant upregulation of the endotoxin tolerance signature in both infected and uninfected M^EP^, but not in M1 or M2 macrophages (Table 1), was in keeping with the identification of M^EP^ macrophages as a distinct phenotype. Furthermore, the dysregulation of this signature (~50% of genes upregulated) in uninfected M^EP^ macrophages indicated that the 24 hr endotoxin priming favoured the induction of an endotoxin tolerance-like phenotype or reprogramming in the M^EP^ macrophages, even prior to a second LPS stimulation. Similarly, uninfected M1 and M2 macrophages upregulated the M1 and M2 signatures [46], respectively (S2 Table). Interestingly, M1 and M2 macrophages but not M^EP^ macrophages upregulated the M1 signature in response to infection with *Salmonella* (S2 Table), indicating that the 24 hr endotoxin priming, as anticipated, resulted in a tolerance response to later stimulation with *Salmonella*. Overall, the expected polarization markers were observed in all three types of macrophages.

### M^EP^ showed resistance to *Salmonella* at the 2 hr time point, but not at the 4 hr and 24 hr time points

*Salmonella* resistance was assessed using a gentamicin protection assay to measure intracellular bacterial load. To determine the dynamics of infection, a time course experiment was performed, taking samples at 0 hr of intracellular infection (immediately after the 30-minute extracellular exposure), 2 hr, and 24 hr. The 0 hr time point quantified initially internalized bacteria, whether this occurred by macrophage phagocytosis or initiated by *Salmonella* Pathogenicity Island (SPI)-1 effectors [47]. The 2 hr time point captured the early stages of infection, just after SCV establishment while the 24 hr time point assessed the ability of *Salmonella* to replicate within the macrophage with the bacterial load reflecting the balance between bacterial killing and replication [47].

**Fig 1:**
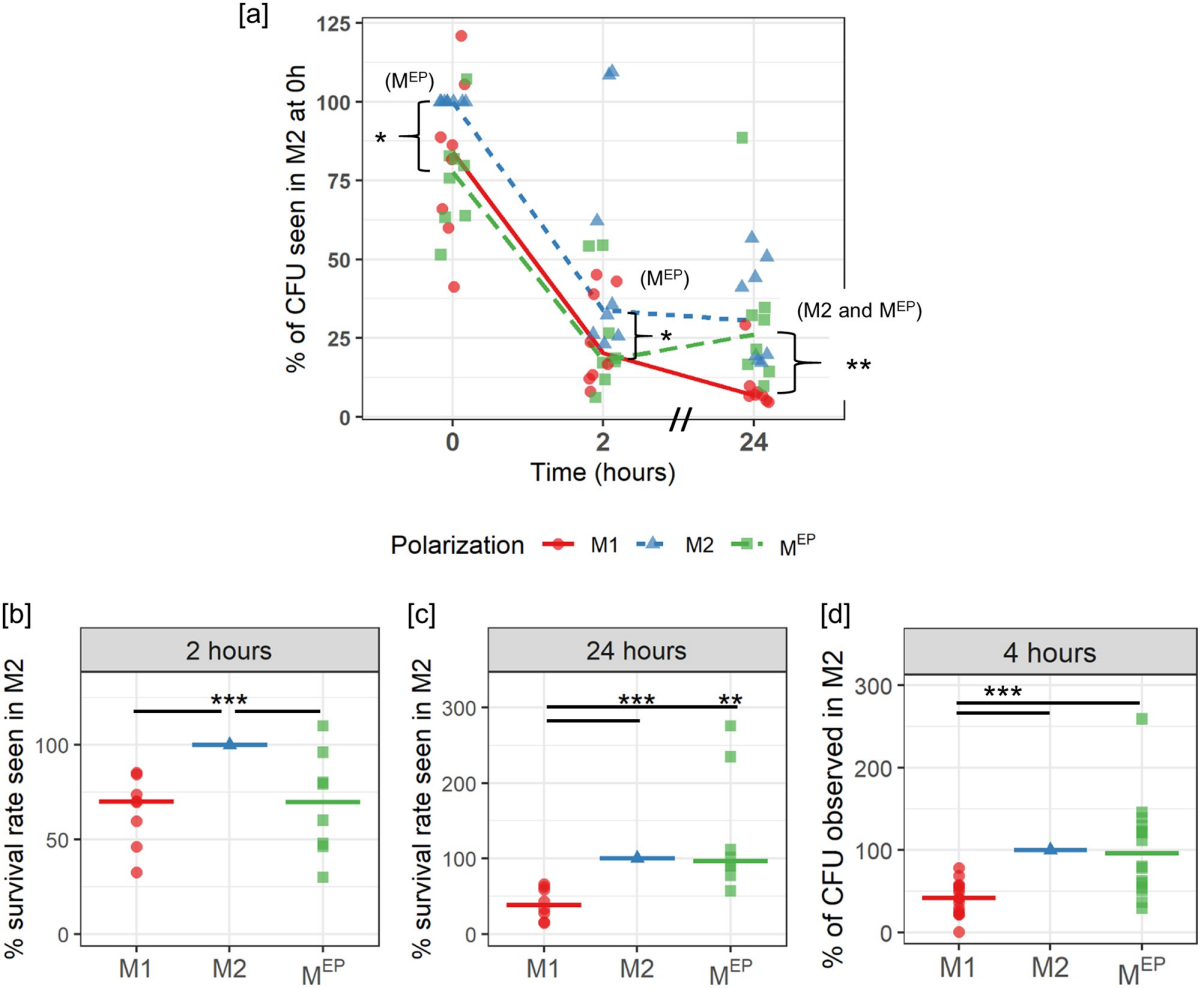
Intracellular bacteria load in polarized cells after the indicated duration of intracellular infection. For each donor, values are expressed relative to the M2 value for that donor. Lines represent the median of all BR. Statistics were calculated by Wilcoxon test, with p-values indicated as * (< 0.05), **(<0.01), ***(<0.001). (a) CFU after 0, 2, and 24 hr intracellular infection (8 BR). 100% represents a median 8.2x10^5^ CFU. (b) Bacterial survival rate from 0 hr to 2 hr, indexed to the M2 survival rate for that donor, median 34%. (c) Bacterial survival rate from 0 hr to 24 hr, indexed to the M2 value for that donor, median 30%. (d) CFU after 4 hr of infection (16 BR). 100% represents a median 2.5x10^5^ CFU.

Interestingly, M^EP^ macrophages showed bacterial loads similar to those of M1 macrophages at both the 0 hr (internalization) and 2 hr time-points (Fig 1a). In M1 and M^EP^ macrophages, a median of 24% and 23% of bacteria survived from the 0 hr to 2 hr time points, compared to a 34% survival rate within M2 macrophages (Fig 1b). In contrast, at 24 hr (Fig 1a), the bacterial load in M^EP^ macrophages was on average 36% of the load observed in M2 macrophages at 0 h, a similar load to that of M2 macrophages (30% of the initial M2 load), while M1 macrophages showed statistically significantly lower levels of bacteria (9.7%). Thus at 24 hours there was a >3-fold higher bacterial load in M^EP^ and M2 than that observed in M1 macrophages (Fig 1a and 1c).

To assess when this switch over occurred, we measured residual intracellular bacteria at 4 hr of infection, observing that both M2 and M^EP^ macrophages were statistically significantly more susceptible to *Salmonella* infection than M1 macrophages, with M1 macrophages showing a median bacterial load of 41.7% of that of M2 macrophages, with M^EP^ macrophages showing a comparable median bacterial load to M2 macrophages (Fig 1d).

These data suggested that, in response to the initial 24 hr stimulation with LPS, the M^EP^ had initially retained some M1 antimicrobial functions, leading to initial resistance to *Salmonella* (Fig 1a and 1b). However the second stimulation likely completed the reprogramming of M^EP^ macrophages such that they had suppressed TNFα expression, failed to upregulate M1 genes, and reduced *Salmonella* resistance (S3 Fig and S2 Table, and Fig 1d). After 24 hr of *Salmonella* infection, there was a further deepened state of immunosuppressive endotoxin tolerance, as indicated by the greater cytokine suppression (S2 Fig) and strong *Salmonella* survival (Fig 1a and 1c). Indeed, repeated doses of LPS are known to increase immune suppression [23, 48].

### M1 and M^EP^ showed primed activation of important anti-infection pathways

To identify possible biological mechanisms explaining differences in *Salmonella* resistance, RNA-Seq analysis was performed on M1, M2, and M^EP^ macrophages. To obtain insights into the loss of *Salmonella* resistance in M^EP^ macrophages, samples were taken at the 4 hr time point, when M^EP^ demonstrated significantly reduced ability to resist *Salmonella* infection (Fig 1d), and the peak of inflammatory responses occurs in M1 macrophages.

**Fig 2:**
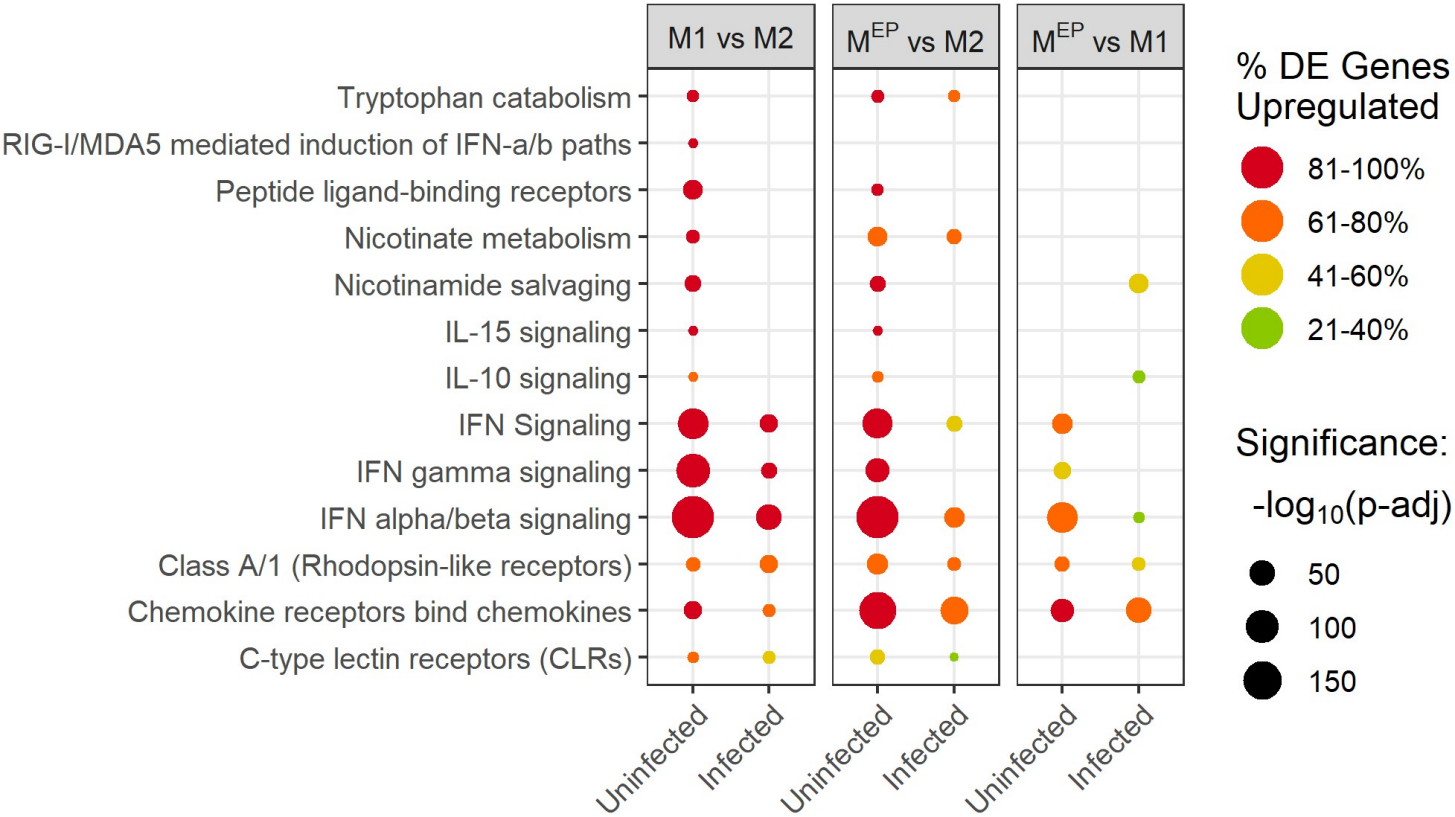
Immune and other pathways showing primed activation. The colour of the dots indicates the percentage of the differentially expressed genes found in the pathway which were upregulated: pathways that were largely upregulated are red, while pathways that were largely downregulated are green. The size of the dots indicates the significance level of the pathway enrichment, -log_10_(p-adj value), such that larger dots have smaller p-values (e.g. 150 refers to the p-value adjusted using the Bonferroni correction for multiple testing, p-adj, of 10^−150^). Only pathways with an adjusted p-value <0.05 are shown.

Pathway enrichment analysis was performed by applying gene-pair signature overrepresentation analysis, *Sigora* [40], to lists of differentially expressed genes derived using *DESeq2* [37]. Several key immune pathways were significantly enriched in uninfected M1 and M^EP^ macrophages, when compared to M2 macrophages (Fig 2).

In contrast, in infected M1 and M^EP^ macrophages, when compared to infected M2 macrophages, these pathways were either not significantly differentially enriched (lack of a spot in Fig 2), or were enriched with larger p-values than in uninfected cells (i.e. the number and pathway specificity of observed gene pairs provide weaker evidence of pathway enrichment; visualized as reduced circle size) or with lower percentages of upregulated genes (visualized as less red colour). Overall, the most significant differences between polarization types for these pathways were in the priming (upregulation of immune genes in the absence of infection) of uninfected macrophages, rather than their response to infection (see S4 Fig for overview). As a result, we refer to this pattern as “primed activation”, which we interpret as meaning that M1 and M^EP^ macrophages were poised to respond to *Salmonella* contact. The increased expression of these genes primarily in uninfected M1 and M^EP^ macrophages suggests that these pathways might have contributed to *Salmonella* resistance in these macrophages through priming of host defence mechanisms prior to contact with the pathogen, allowing these defenses to take effect during the initial stages of *Salmonella* uptake and growth. Conversely, the loss of resistance in M^EP^ after 4 hr likely resulted from differences in other pathways, as described below. In contrast, M2 macrophages only upregulated these genes in the presence of *Salmonella*. Despite reaching similar expression levels in these pathways when infected, M2 macrophages were not able to resist *Salmonella*, possibly as a result of a delayed immune response.

The primed activation pathways included several pathways known to be involved in intracellular infection, such as interferon pathways [49] labelled as “interferon (IFN) signaling”, “IFNγ signaling”, “IFNα/β signaling”, “RIG-I/MDA5 mediated induction of IFNα/β”; and “C-type Lectin Receptors (CLRs)”, which are important in defences against viruses, and likely intracellular bacterial pathogens like *Salmonella* [50]. Additional primed activation immune pathways included “Class A/1 Rhodopsin-like receptors”, many of which are involved in inflammatory signalling, “Chemokine Receptors bind Chemokines”, “IL-10 signaling”, and “IL-15 signaling”. When compared to uninfected M2 macrophages, both uninfected M1 and M^EP^ macrophages highly upregulated chemokine CXCL11/I-TAC that binds to the CXCR3 receptor, which is known to be an important component in the host defense against *Salmonella* [51]. IL-10 is induced by TNFα in a negative feedback loop to limit inflammation, and protects macrophages from TNFα-induced apoptosis during *Salmonella* infection [52]. IL-15 has been implicated in *Salmonella* killing via activation of natural killer cells [53] and also is produced by monocytes and influences infection and inflammation [54]. It is interesting that these pathways related to intracellular infection and *Salmonella* were active in uninfected M1 macrophages, while such changes were not observed in the TLR signaling pathways that control general infection and inflammation.

In addition, three pathways connected to metabolism demonstrated primed activation, namely “Tryptophan catabolism” (e.g. IDO1), “Nicotinate metabolism” (e.g. CD38) and “Nicotinamide salvaging” (e.g. NAMPT). These pathways are linked since they all represent routes for production of NAD^+^, which has a variety of effects on immunity [55]. In particular, levels of extracellular NAD^+^ mobilize monocytes and neutrophils through the activity of CD38 [56], and CD38 activation interferes with *Salmonella*’s ability to invade macrophages [57], while increased NAD^+^ synthesis during infection can be protective [55, 56], and may prevent free radical damage during oxidative killing [58]. Thus primed activation of NAD^+^ producing pathways in uninfected M1 and M^EP^ macrophages might prepare them for a stronger oxidative response to *Salmonella* infection, increasing killing capacity. IDO1 has been further implicated in defense against *Chlamydia* [59] as well as in *Salmonella* killing, due to either nutrient restriction or the immunomodulatory properties of Trp catabolites [58]. The observation of these pathways in uninfected M1 macrophages is thus consistent with the proposition that M1 macrophages are metabolically non-permissive for *Salmonella* replication [60]; that is they were inhospitable to the pathogen even before infection occurred.

### JAK-STAT genes were a central component of the primed activation network for M1 macrophages

**Fig 3:**
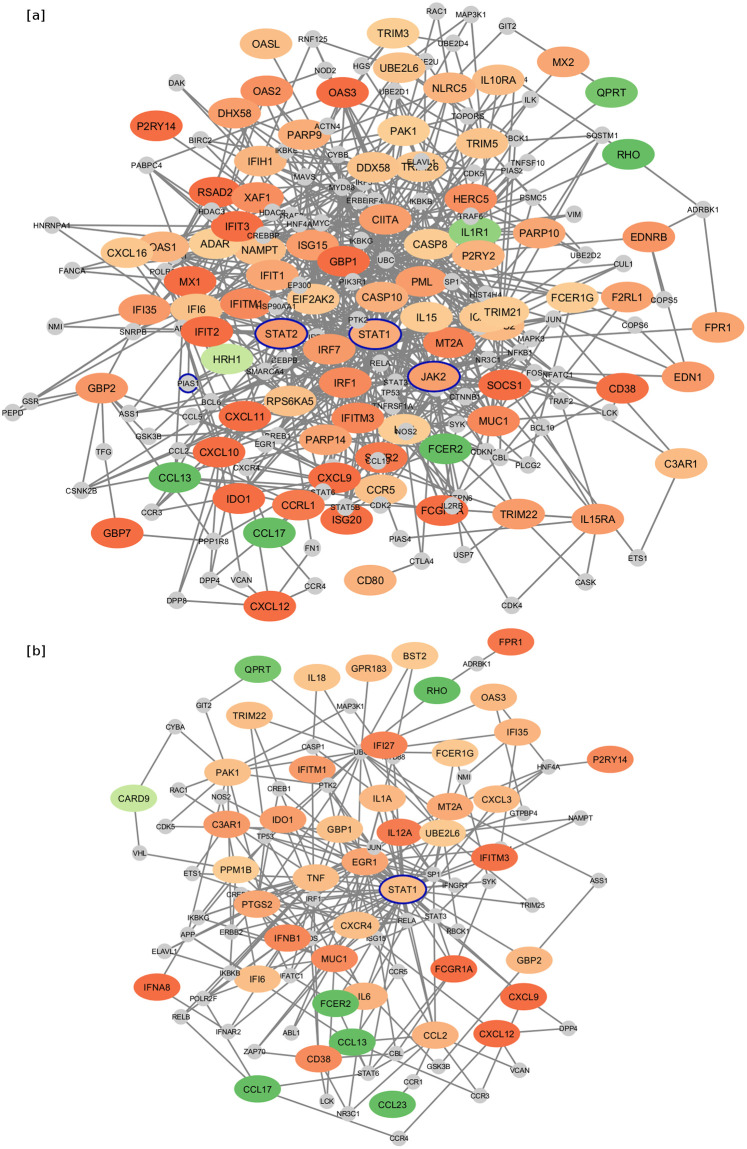
Minimal first order protein:protein interaction networks of genes in primed activation pathways. Comparing (a) uninfected M1 vs. uninfected M2 and (b) infected M1 vs infected M2 macrophages. Red-orange genes are upregulated while green genes are downregulated, with darker shades indicating a greater fold change. Smaller grey circles indicate genes that were not differentially expressed, but connect two or more differentially expressed genes in the network. Genes of interest STAT1, STAT2, JAK2, and PIAS1 are outlined in blue for emphasis.

Protein:protein interaction networks are a useful tool for providing visual depiction of differentially expressed genes, based on the known (function-based) interactions between the protein products of these genes [30, 42]. Proteins are represented as nodes (ovals in Fig 3), while interactions between the proteins are represented as “edges” connecting two nodes (lines in Fig 3). Central or “hub” nodes can be identified by hub degree, which is the number of connections with other nodes/proteins. Minimally connected networks (Fig 3) were created from the list of genes that were differentially expressed in M1 vs. M2 macrophages, and were part of the primed activation pathways indicated in Fig 2. Among the genes from primed activation pathways that were upregulated in uninfected M1 when compared to M2 macrophages (Fig 3a), transcription factor STAT1 was particularly prominent with the highest hub degree of 63. STAT2 and JAK2 were also represented with degrees of 25 and 23, respectively. When comparing infected M1 and M2 macrophages (Fig 3b), STAT1 still had the highest hub degree of 38, but STAT2 and JAK2 were no longer differentially expressed.

When considering the primed activation of JAK-STAT signaling as a possible mechanism of resistance, it is interesting to note that JAK-STAT signaling participants were primarily upregulated in uninfected M1 macrophages, while JAK-STAT effectors were more upregulated in infected M1 macrophages. This indicated that JAK-STAT signaling might be a central component of the primed activation response, with priming of the overall pathway leading to an elevated upregulation of specific effector genes in M1 macrophages upon encountering *Salmonella*. Infected M1 still expressed higher levels of STAT1 than infected M2 macrophages, and more JAK2 than M^EP^ macrophages, but otherwise their differentially expressed JAK-STAT pathway genes largely represented effectors such as IFNA8, IFNW1, IL-12A, and IL-6, which are also regulated by many other pathways.

JAK-STAT signaling is a major mechanism through which cytokine signaling is translated into immune responses, including during macrophage activation [61]. Thus suppression of JAK-STAT signaling mRNA is associated with *Salmonella* susceptibility in chickens [62], and *S*. *enterica* serovar Enteritidis inhibits JAK-STAT signaling in chickens to subvert host defenses [63]. Notably, the IFNγ-induced expression of Guanylate Binding Proteins, which rupture the SCV, is dependent on JAK/STAT signalling [7]. Also persistent *Salmonella* infection is associated with dephosphorylation / deactivation of JAK2 [63]. Furthermore, STAT1 deficient mice are defective in immunity to intracellular bacteria [64] and STAT1 deficiency or mutation in humans is associated with susceptibility to *Salmonella* infection [65]. Also STAT2 is known to be expressed in response to *Salmonella* infection [11] and STAT2-dependent inflammation in the gut produces a competitive advantage for *Salmonella* over the gut microbiota, leading to *Salmonella* growth [66]. Thus these pathways can be strikingly associated with the fate of *Salmonella* infections.

Additional links between this network of primed JAK-STAT genes and *Salmonella* can be found in a recent study that identified genes for which a loss of function mutation resulted in *Salmonella* resistance in macrophages [67]. Among these genes is PIAS1 (Protein Inhibitor of Activated STAT 1), which is a connecting gene of degree 6 in the primed activation network for uninfected M1 and M2 macrophages (Fig 3a), but is absent from the network for infected M1 and M2 macrophages (Fig 3b). Since knocking out this STAT1 inhibitor gene within this primed activation network results in *Salmonella* resistance, it follows that the network and primed activation of JAK-STAT is important to resistance.

### Inflammasomes were enriched in infected M1 macrophages, but not infected M^EP^ macrophages

Inflammasomes are notable as a major *Salmonella*-defensive pathway that did not exhibit a primed activation pattern. Inflammasomes are multi-protein complexes that trigger inflammatory cell death (pyroptosis) and the processing and release of IL-1β and IL-18 [68], activating immune responses [68] and exposing *Salmonella* to killing by neutrophils [69]. This process is believed to be important in detecting and defending against intracellular *Salmonella* infection, since mice lacking the pyroptotic caspase-1, IL-1β, or IL-18 are more susceptible to *Salmonella* [70].

**Fig 4:**
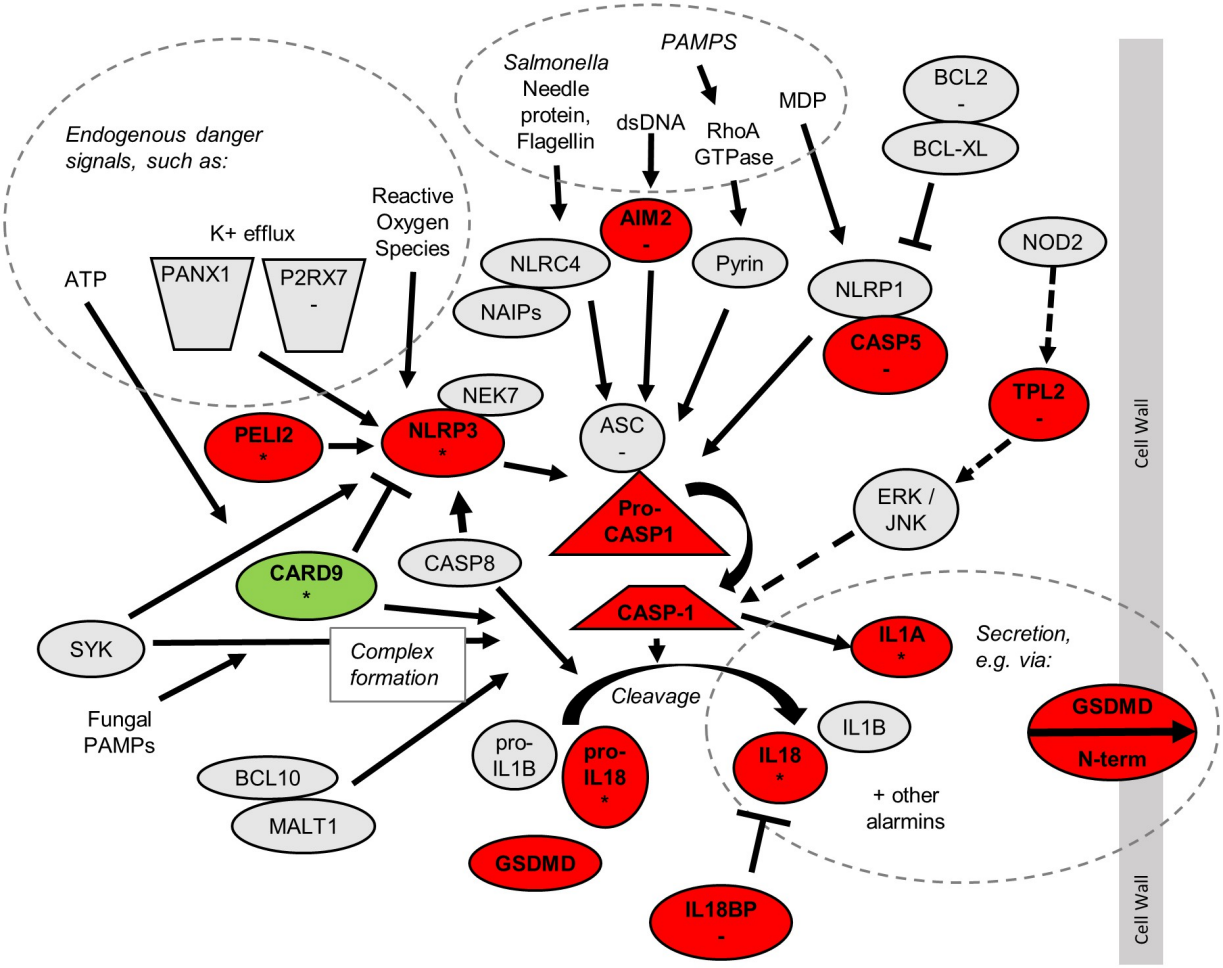
Inflammasome signaling pathways showing genes dysregulated between differently polarized MDM that are infected with *Salmonella*. Genes upregulated in infected M1 vs. infected M2 macrophages are in red. Genes downregulated in infected M1 vs. infected M2 macrophages are in green. Genes that dysregulated in M^EP^ compared to M2 macrophages in the same direction as in M1 vs. M2 macrophages are indicated with “*”. Genes downregulated in M^EP^ vs. M1 macrophages are indicated with “-”.

Compared to infected M2 macrophages, infected M1 macrophages upregulated 10 inflammasome genes, including key genes at multiple levels of the inflammasome process (Fig 4). These included initiating receptors AIM2 and NLRP3, facilitator PELI2, central pyroptosis coordinator CASP1 (caspase-1), pyroptosis mediator Gasdermin-D (GSDMD) and effector cytokines IL-1A and IL-18, as well as MAP3K8 (TPL2), which is required for IL-1β secretion in response to various PRR agonists and *Salmonella* [71]. Similarly, infected M1 macrophages downregulated CARD9, which inhibits the NLRP3 inflammasome during *Salmonella* infection [72]. Upregulation of the NLRP3 inflammasome could provide a route for detection of *Salmonella* even if *Salmonella* evades detection by NLRC4 as a result of suppressing flagellin [73, 74]. In response to infection, M1 macrophages showed a greater gene expression of two downstream effectors of inflammasome signaling, IL-1α and IL-18, than did M2 macrophages, but IL-1β was not significantly upregulated in infected M1 macrophages.

The lack of primed activation was shown by the observation that uninfected M1 macrophages upregulated 9 inflammasome genes (S5 Fig), which did not include NLRP3, PELI2, or CASP1, and did not downregulate the CARD9 inhibitor.

Infected M^EP^ macrophages showed higher expression of 6 inflammasome genes when compared to M2 macrophages, including NLRP3 and IL-18, but did not show a relative difference in IL-1A and IL-1B expression in response to infection. Consistent with the reduced effect of endotoxin priming on inflammasome genes, M^EP^ macrophages showed significantly lower expression of 8 inflammasome genes including receptor AIM2, complex component ASC, and CASP5, when compared to infected M1 macrophages. Furthermore, M^EP^ macrophages downregulated receptor IL-1R1 and accessory IL-1RAP when compared to M2 macrophages, consistent with decreased inflammasome-mediated defenses. The low number of upregulated inflammasome genes in M^EP^ macrophages infected with *Salmonella* is consistent with and likely reflected and/or explained the tolerance of these cells to *Salmonella* infection.

Overall, these data suggest that when exposed to *Salmonella*, M1 macrophages increased their expression of genes involved in inflammasome formation and pyroptotic signaling, which would promote resistance to infection. Lower expression of these genes, and especially downstream effectors could contribute to the lack of similar resistance in M2 macrophages, and in M^EP^ macrophages after 4 hr.

### *Salmonella*-associated genes were differentially regulated as a result of polarization

Recently CRISPR methods were utilized to identify a set of 183 genes for which a loss of function mutation resulted in reduced *Salmonella* infection of macrophages [67]. Thus, downregulation of these genes would appear to favour *Salmonella* resistance. Differential enrichment in this gene-set was assessed using *roast* (Table 2). Intriguingly these susceptibility genes were overall downregulated in infected M1 macrophages cf. M2 macrophages, consistent with the resistant status of M1 cells. Conversely this gene-set was relatively upregulated in infected M^EP^ cf. M1 macrophages (Table 2), consistent with the ability of *Salmonella* to grow in M^EP^ macrophages after 4 hr.

**Table 2:** *Roast* gene-set enrichment test for a set of 183 susceptibility genes [67] and a set of 31 *Salmonella* targets and SCV-implicated genes (S1 Table).

MDM Types Compared	*Salmonella* susceptibility gene-set				*Salmonella* targets and SCV-implicated genes			
	% Up^1^	% Down^1^	Direct-ion^2^	p-val^3^	% Up^1^	% Down^1^	Direct-ion^2^	p-val^3^
M1 vs. M2 uninfected	17	16	Up	0.88	27	4	Up	0.40
M^EP^ vs. M1 uninfected	31	15	Up	0.31	12	23	Down	0.30
M^EP^ vs. M2 uninfected	30	19	Up	0.27	23	27	Down	0.75
M1 vs. M2 infected	**5.8**	**33**	**Down**	**0.036**	0	31	Down	0.064
M^EP^ vs. M1 infected	**51**	**9.1**	**Up**	**<0.001**	38	12	Up	0.068
M^EP^ vs. M2 infected	41	19	Up	0.074	31	35	Up	0.96
M1 infected vs. uninfected	**8.3**	**68**	**Down**	**<0.001**	**0**	**77**	**Down**	**<0.001**
M2 infected vs. uninfected	**7.5**	**52**	**Down**	**0.001**	**4**	**46**	**Down**	**0.007**
M^EP^ infected vs. uninfected	**6.7**	**38**	**Down**	**0.013**	**0**	**38**	**Down**	**0.017**

^1^ Percentage of the genes in the signature that were found to be up- or down-regulated for the comparison in question by *roast*

^2^ Whether the signature was found to be overall up-or down-regulated

^3^ p-value was calculated by *roast* taking into account both percentage of up- and down-regulated genes and magnitude of change. Since 99999 rotations were performed the minimal p value is 0.001; Statistically significant (p<0.05) comparisons are bolded.

Interestingly, the expression of the susceptibility gene-set was not statistically significantly altered in any uninfected macrophage type, although the set was significantly downregulated in all macrophage types in response to infection (Table 2). The downregulation of this susceptibility gene-set upon infection was largest in M1 macrophages, with 68% of these genes downregulated in infected M1 macrophages compared to uninfected M1 macrophages, while only 52% were downregulated upon infection of M2 macrophages and 38% downregulated in M^EP^.

*Salmonella* manipulates a variety of genes in order to suppress host defenses and/or to promote SCV formation and/or intracellular infection; this manipulation is important to *Salmonella*’s intracellular survival [75]. A set of 31 *Salmonella* target genes and genes involved in SCV formation was obtained by augmenting a BioCarta gene list with additional findings from the literature (S1 Table). As with the susceptibility gene-set, the *Salmonella*-targets gene-set was downregulated in each of M1, M2, and M^EP^ macrophages in response to infection (Table 2), with the greatest downregulation occurring in M1 macrophages (77% of genes in this set downregulated, p-value <0.001) and the lowest downregulation in M^EP^ macrophages (38% downregulated, p-value 0.017). The most downregulated *Salmonella*-target genes in M1 macrophages in response to infection were VPS18 (FC -3.68), PLEKHM1 (FC -2.53), and EEA1 (FC -2.28).

### Pathways upregulated in M^EP^ compared to M1 included the metallothioneins and chemokine receptors

**Fig 5:**
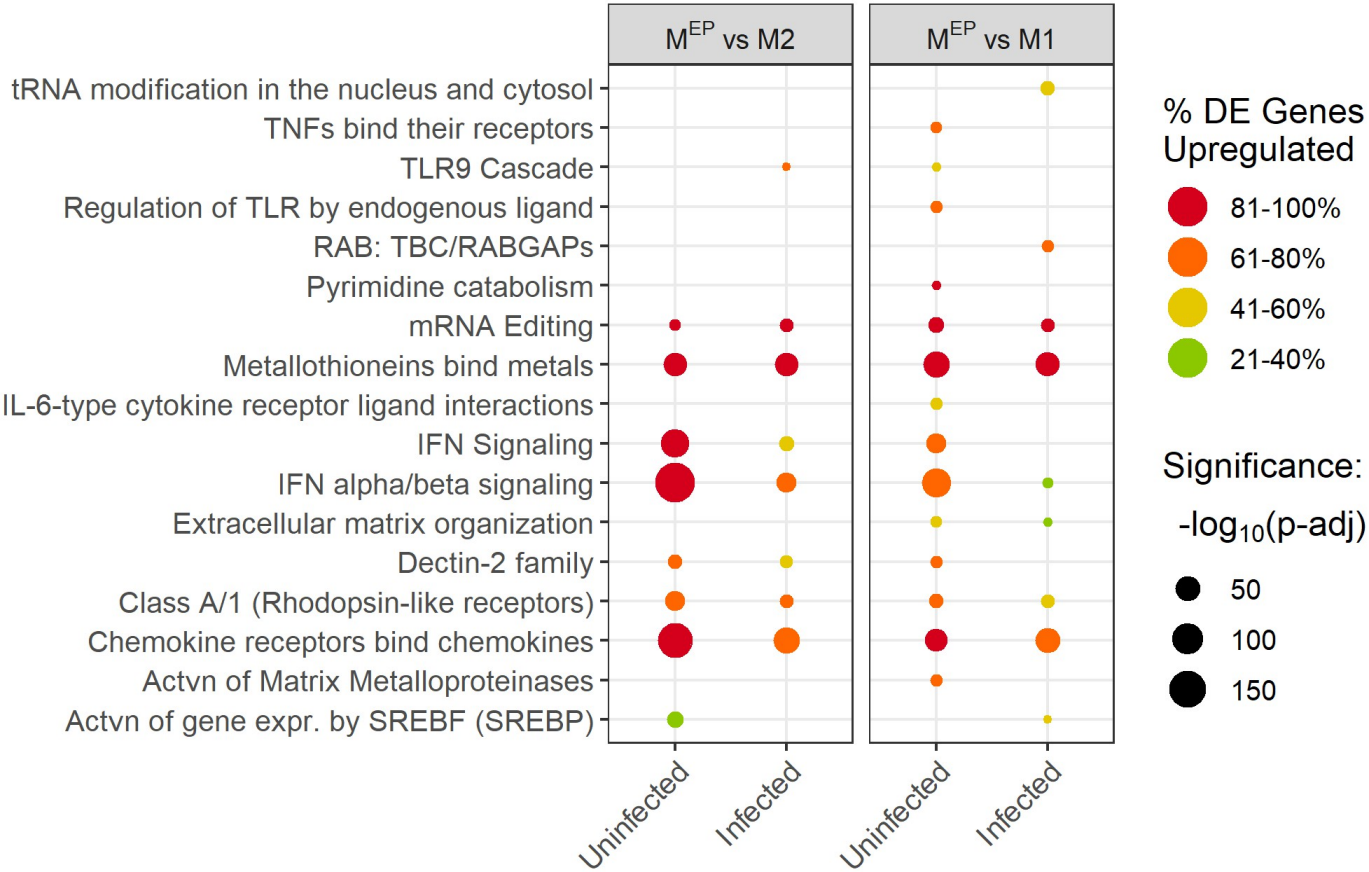
Pathways upregulated in in either uninfected or infected M^EP^ when compared to M1 macrophages. The colour of the dots indicate the percentage of the differentially expressed genes found in the pathway which were upregulated: pathways that were largely upregulated are red, while pathways that were largely downregulated are green. The size of the dots indicates the significance level of the pathway enrichment, -log_10_(adjusted p-value), such that larger dots have smaller p-values. “p-adj” refers to the p-value adjusted using the Bonferroni correction for multiple testing. Only pathways for which the adjusted p-value was ≤0.05 are shown.

Since only a small number of pathways were upregulated in M^EP^, when compared to M1 macrophages in either infected or uninfected cells, these pathways were further examined to identify the pathways characteristic of M^EP^ macrophages (Fig 5).

These M^EP^-upregulated pathways included “chemokine receptors bind chemokines”, and “Metallothioneins bind metals”, both of which were upregulated in M^EP^ macrophages regardless of infection status. The upregulation of the metallothionein pathway was consistent with their inclusion in the endotoxin tolerance signature (Table 1). Metallothioneins have been proposed to increase bacterial clearance by macrophages [76], but any effect on the initial resistance of M^EP^ macrophages did not last beyond the 2 hr time point. Regarding chemokines, uninfected M^EP^ macrophages expressed high levels of CXCL11 and CCL5/RANTES, which are ligands of CXCR3 an important receptor in defence against *Salmonella* [51], but high expression of these chemokine genes was not observed in infected M^EP^. Chemokines upregulated in both infected and uninfected M^EP^ included the neonatal sepsis biomarker, lymphocyte-attracting CXCL12/SDF-1 [77], and neutrophil-attracting chemokines CXCL5/ENA-78 [78] and CXCL6/GCP2, as well as B lymphocyte chemoattractant CXCL13/BLC. Although chemokine induction has been suggested to have a protective role (e.g. [51]), it has also been suggested that gut inflammation—resulting from induction of chemokines [79] including CXCL6 [80]–provides *Salmonella* with a competitive advantage relative to the host microbiota [81].

Macrophage responsiveness to TLR ligands and interferons, and establishment of endotoxin tolerance, depend on a variety of epigenetic modifications [82]. We examined transcriptional changes in a set of 70 histone deacetylases, DNA methyltransferases, histone methyl transferases, and other genes implicated in the literature in epigenetic modification during macrophage activation (S3 Table). Of these, 23 were differentially expressed under at least one condition studied. Most of these genes were up-or down-regulated in response to infection, with few differences between macrophage activation types (S6 Fig).

As expected, IRF1 was upregulated in uninfected M1 macrophages compared to both M^EP^ and M2 macrophages; this transcription factor is known to be induced by type I and II interferons, and is associated with chromatin modifications that counteract endotoxin tolerance [83]. Infected M^EP^ showed a modest (< 4-fold) upregulation in SMYD4, SETD5, NSD1, and EZH1 compared to both infected M1 and M2 macrophages, and infected M^EP^ showed a downregulation in SMYD2, KDM6B/JMJD3, and HDAC4 compared to infected M1 macrophages. To our knowledge, there is no clear association between these genes and endotoxin tolerance, although EZH1 promotes inflammatory signalling in macrophages [84] and SMYD2 is a negative regulator of M1 polarization [85], while JMJ3D is associated with establishment of M2 activation [86]. Overall, we conclude that any epigenetic changes involving macrophage activation are by-and-large not regulated at the transcriptional level.

### Inhibition of JAK using Ruxolitinib increased M1 susceptibility to infection

As described above, JAK2, STAT1, and STAT2 are key hubs in the M1 macrophage network for certain key immune pathways are upregulated or primed in uninfected M1 macrophages. These observations led us to hypothesize that priming of JAK-STAT signaling pathways as a result of polarization led to more efficient expression of their effectors upon introduction of *Salmonella*, resulting in *Salmonella* resistance in M1, but not in M2, or likely M^EP^, macrophages. To test this hypothesis, the JAK1/2 inhibitor Ruxolitinib was applied to macrophages during the polarization phase, rather than the infection phase, to suppress any effects of increased JAK-STAT expression occurring prior to infection but not preventing altered expression of these genes in response to infection.

**Fig 6:**
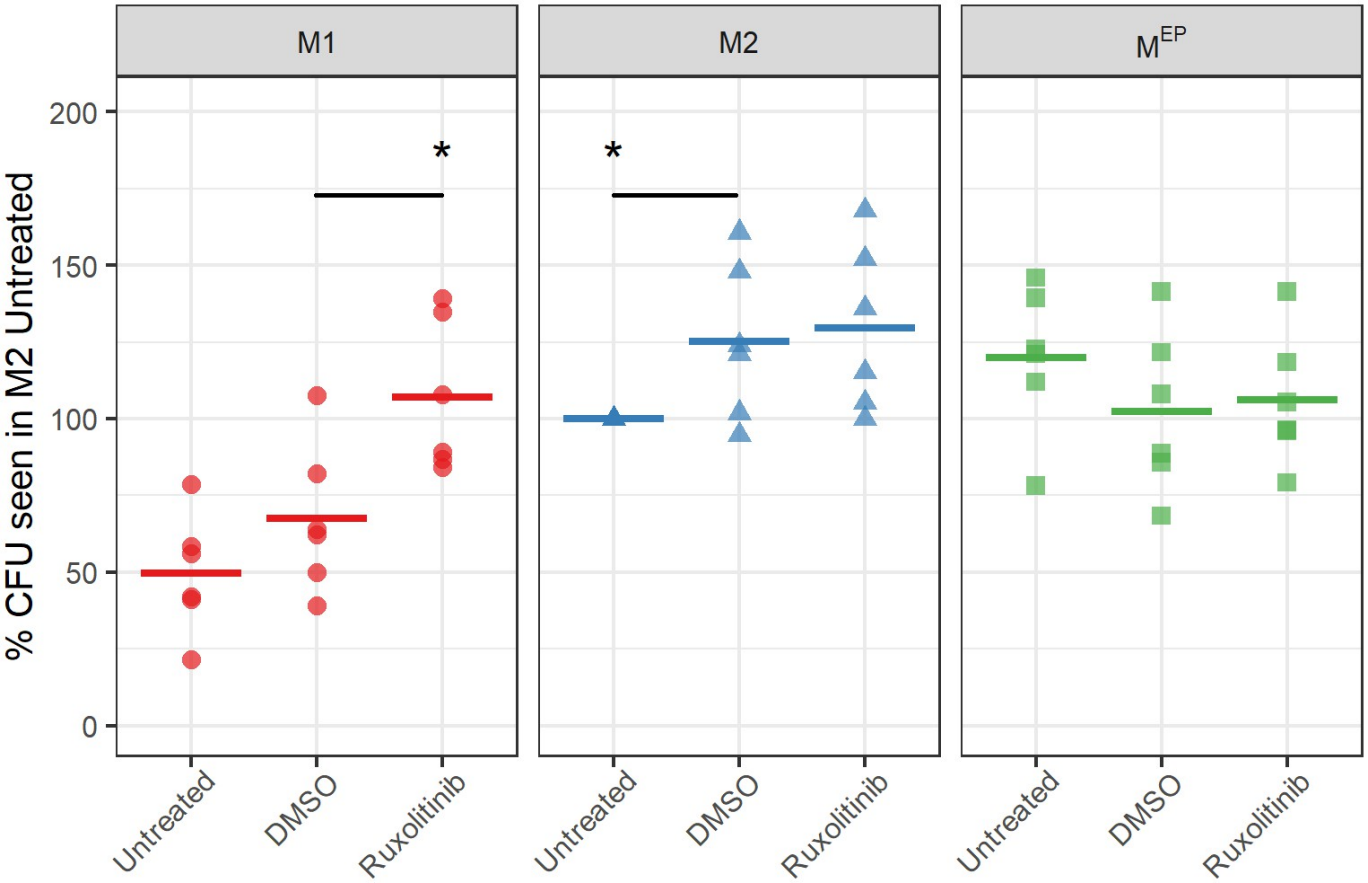
Effect of JAK inhibition during polarization on *Salmonella* resistance. Color and shape reflect polarization states as labelled and is used for emphasis. For each donor, data were expressed relative to CFU in untreated M2 macrophages from the same donor; lines represent the medians of 6 biological replicates (BR). 100% reflects a median 3.4x10^7^ CFU. Significance was determined relative to the DMSO control for each polarization state. Statistics were calculated by Wilcox test, with p-values indicated as * (< 0.05).

**Fig 7:**
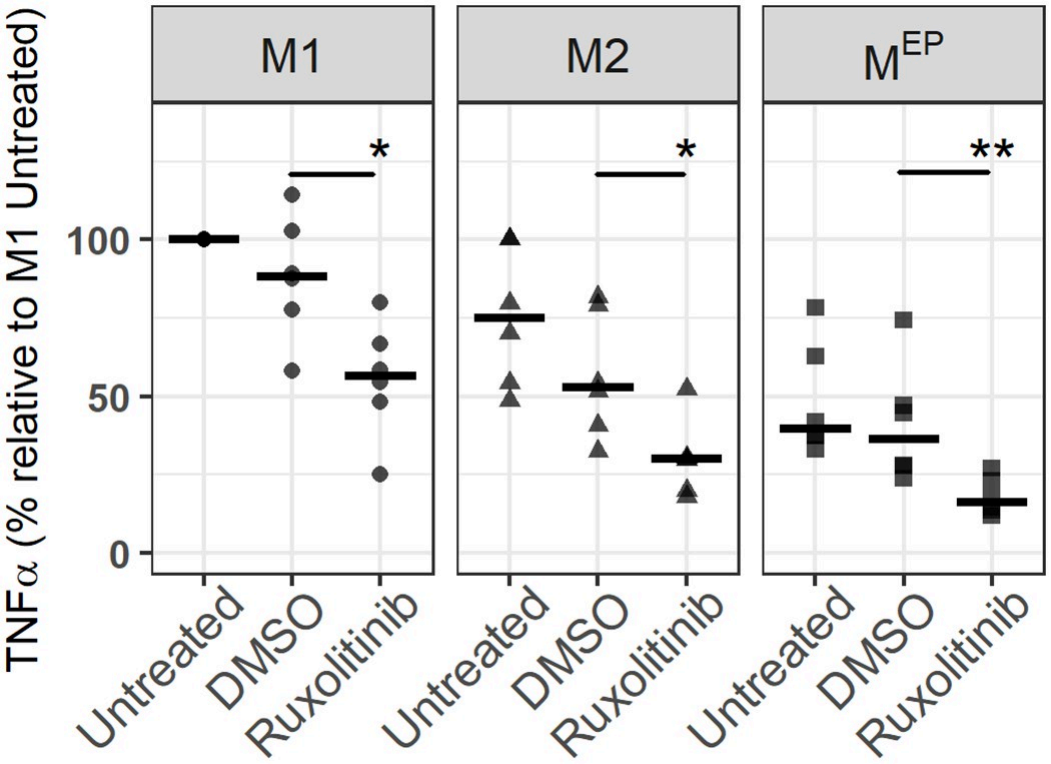
Effect of JAK inhibition on TNFα expression measured after infection. For each donor, data was expressed relative to the expression level in the untreated M1 macrophages from the same donor. 100% represents a median of 87 ng/mL TNFα. Lines represent the median of 6 BR. Significance is determined relative to the DMSO control for each polarization state as calculated by Wilcox test, with p-values indicated as * (< 0.05), **(<0.01).

When JAK1 and JAK2 were inhibited using Ruxolitinib during the polarization phase, the *Salmonella* load in M1 macrophages increased significantly by 60% when compared to the DMSO control (Fig 6) to a level comparable to that of untreated M2 macrophages. In contrast, no increase in the bacterial load in M2 (or M^EP^) macrophages was observed as a result of JAK inhibition. The DMSO vehicle caused modest changes in bacterial load in both M1 and M2 macrophages. This was consistent with the hypothesis that *Salmonella* resistance observed in M1 macrophages, relative to M2 macrophages, resulted at least in part from primed activation of JAK-STAT signalling, prior to *Salmonella* exposure. To demonstrate that the inhibitor was working we showed that it inhibited TNFα production in all 3 macrophage types after infection (Fig 7).

## Conclusions

While polarization is known to have an effect on the resistance of macrophages to *Salmonella* infection [11, 12], the effect of endotoxin priming on the resistance of MDM was previously undetermined, and a whole-transcriptome comparison of the response of differently polarized MDM to infection had not been performed. The current study provided the first determination of *Salmonella* resistance and susceptibility in endotoxin primed (M^EP^) MDM, which is relevant given the association of such macrophages with sepsis and the frequent death of sepsis patients from secondary infections. We also presented a comprehensive picture of differing transcriptional responses in M^EP^, M1 and M2 macrophages to *Salmonella* infection, identified likely mechanisms for differing resistance to the pathogen, and provided evidence that JAK-STAT signaling is mechanistically important for the response of M1 macrophages to *Salmonella* infection, but not for M2 or for M^EP^ macrophages at the 4 hr time point.

Intriguingly, 24 hr endotoxin priming of macrophages initially resulted in resistance to *Salmonella* during the first 2 hr of infection. However, the cellular reprogramming associated with tolerance rapidly resulted in a failure to engage specific defensive mechanisms in response to *Salmonella* infection, causing M^EP^ macrophages to be susceptible to *Salmonella* at both the 4 hr and 24 hr time points. This finding has important implications when considering that, while the risk of mortality in early stages of sepsis derives from high inflammation and organ failure, mortality in later stages results from immune suppression [19]. Specifically, the inability of M^EP^ macrophages to induce inflammatory responses would encourage subsequent infections even through the M1-like early killing suggests the possibility that certain antimicrobial defenses might have been preserved. The inability to control replication of *Salmonella* beyond 4 hr of exposure is consistent with the known profound and deadly immunosuppression associated with late-stage sepsis [19] and increase in secondary infections [87]. Targeting the mechanisms leading to the loss of resistance to *Salmonella* in endotoxin primed macrophages could form the basis of immunomodulatory treatments for sepsis.

Our transcriptional data suggested that M1 macrophage resistance to *Salmonella* infection likely results from a combination of at least three elements. First, primed activation of key immune pathways and genes, including JAK-STAT signaling, likely reflected an improved ability to mount a rapid immune response to *Salmonella*, in contrast to M2 macrophages that activated these pathways only in response to *Salmonella* infection. Second, upregulation of inflammasomes and particularly the NLRP3 inflammasome in response to infection might contribute to anti-infective pyroptosis. Third, downregulation of *Salmonella* target genes and other susceptibility genes could interfere with *Salmonella*’s ability to manipulate the cytoskeleton, etc., to promote SCV survival. The observation of primed activation in tryptophan and nicotinate pathways may further indicate a significant role for NAD^+^ in *Salmonella* infection, as suggested previously [55, 56], and a novel importance for this pathway in M1 macrophages. It is worth mentioning however that this systems biology examination of *Salmonella* infection has demonstrated numerous pathways/mechanisms with known supportive and inhibitory functions on such infections, and thus it seems likely that it is the overall integration of these pathways that critically determines the fate of *Salmonella*.

For M^EP^ macrophages, the difference in bacterial killing at the early 2 hr and later 24 hr time points likely reflects differences between M1 and M^EP^ macrophage activation. Uninfected M^EP^ macrophages demonstrated certain pathways that were primed and/or activated in a manner similar to M1 macrophages. Given that these pathways were induced in the absence of *Salmonella*, they were probably active during the earlier phases of infection and contributed to early killing of *Salmonella*. In keeping with this idea that genes differentially expressed in uninfected MDM might contribute to early resistance, uninfected M^EP^ macrophages showed a similar expression of *Salmonella* susceptibility and target genes (Table 2) when compared to uninfected M1 macrophages. However, greater differences were observed in activated genes and pathways when comparing infected M^EP^ with infected M1 macrophages. In particular, infected M^EP^ macrophages showed lower expression of inflammasome genes than did infected M1 macrophages, while M^EP^ macrophages demonstrated downregulation of fewer *Salmonella* target and susceptibility genes when compared to M1 macrophages. In combination, these transcriptional effects could have been responsible for the increased intracellular *Salmonella* persistence in M^EP^ macrophages as the infection progressed.

Additional work could also investigate the effect of endotoxin priming over longer periods of time. During *Salmonella* infection, intracellular replication persists for several days [1], and immunosuppression resulting from sepsis can also be very long-lasting [19]. Therefore, it would be of interest to determine whether the ultimate susceptibility of M^EP^ to *Salmonella* infection could persist if the infection occurred one or more days after the initial tolerance-inducing endotoxin priming. Such studies are ongoing in our lab.

The described mechanisms identified through the transcriptomic analysis provide potential targets for immunomodulatory treatments of *Salmonella* infections. However, this requires that these mechanisms are verified experimentally. In keeping with the hypothesized importance of primed activation of JAK-STAT genes in M1 resistance, inhibition of JAK1/2 by Ruxolitinib treatment during the polarization phase (prior to infection) resulted in the increased susceptibility of M1 macrophages to *Salmonella*. We have not verified activation of JAK-STAT at the protein level. Further work building on this transcriptomic study should confirm that levels of phosphorylated p-JAK are higher in uninfected M1 than M2 macrophages. Moreover, it would be interesting to determine which of the effectors downstream of JAK were responsible for inducing resistance in M1 macrophages and thus would represent potential targets for intervention.

## Supporting information

S1 Table Gene-sets used for *roast* analysis: Polarization and endotoxin tolerance signatures, and known *Salmonella* susceptibility genes. Gene-set for endotoxin tolerance was taken from Pena *et al* (https://doi.org/10.1016/j.ebiom.2014.10.003). Gene-set for polarization was taken from Becker et al (https://doi.org/10.1038/srep13351). Gene-set for *Salmonella* susceptibility was taken from BioCarta (h_salmonellaPathway) (https://doi.org/10.1089/152791601750294344), as collated in the Molecular Signatures Database (MSigDB.v6.2) (https://doi.org/10.1073/pnas.0506580102; https://doi.org/10.1093/bioinformatics/btr260), supplemented with genes identified in the literature, as listed in the final column of the table.
(CSV)

S2 Table *Roast* gene-set enrichment test results for M1 and M2 signature genes. The M1 and M2 gene signatures were derived by Becker *et al* (https://doi.org/10.1038/srep13351).
(XLSX)

S3 Table Genes involved in chromatin modification or other epigenetic regulation. Column “Reference” indicates reference identifying the gene as involved in macrophage modification, if applicable. If no reference is given, the gene is generally known to be a histone deacetylase, DNA methyltransferase, or histone methyl transferase.
(CSV)

S4 Table Gene expression changes calculated by DESeq2 for MDM. Comparisons are: infected versus uninfected MDM for each of M1, M2, and M^EP^ polarization (M1/M2/MEP_IvU), M1 versus M2 for uninfected and infected MDM (M1vM2_U and M1vM2_I respectively), M^EP^ versus M2 for uninfected and infected MDM (MEPvM2_U and MEPvM2_I), and M^EP^ versus M1 for uninfected and infected MDM (MEPvM1_U and MEPvM1_I).
(XLSX)

S1 Fig PCA plot showing clustering of normalized gene expression data for differently activated MDMs that were uninfected or infected with *Salmonella*. The first two principal components, plotted on the X and Y axes, summarize the greatest sources of variation between samples.
(PNG)

S2 Fig Cytokine production in polarized MDM infected with *Salmonella*, after 24 hr intracellular infection. Colour corresponds to polarization, and is used for emphasis. Data is from 8 biological repeats and expressed relative to cytokine expression in M1 macrophages for the same donor; 100% represents an average 35,900pg/mL TNFα, 91,000pg/mL IL-6, 1,750pg/mL IL-10, and 345pg/mL IL-1β. Statistics were calculated by Wilcoxon test, with p-values indicated as * (< 0.05), ** (<0.01), *** (<0.001).
(PNG)

S3 Fig Cytokine production in polarized MDM which were uninfected or infected with *Salmonella*, after 4 hr intracellular infection. For each donor, production is expressed relative to the level observed in infected M1 macrophages; 100% represents an average 67,300pg/mL TNFα, 57,300pg/mL IL-6, 237pg/mL IL-10, 201pg/mL IL-1β, and 7,640pg/mL MCP-1. Colour corresponds to polarization, and is used for emphasis. Data represents 4 biological replicates for uninfected cells, and for infected cells 16 biological repeats for TNFα, 13 BR IL-6 and IL-10, 9 BR IL-1β, 6 BR MCP-1. Statistics were calculated by Wilcoxon test, with p-values indicated as * (< 0.05), ** (<0.01), *** (<0.001).
(PNG)

S4 Fig RNA-Seq comparisons relevant to primed activation. (PNG)

S5 Fig Inflammasome signaling pathways showing genes dysregulated between differently polarized MDM that were not infected with *Salmonella*. Genes upregulated in uninfected M1 vs. uninfected M2 macrophages are in red. Genes downregulated in uninfected M1 vs. uninfected M2 macrophages are in green. Genes up or downregulated in M^EP^ vs. M2 macrophages are indicated with “u” and “d” respectively. Genes up or downregulated in M^EP^ vs. M1 macrophages are indicated with “+” and “-” respectively.
(PNG)

S6 Fig Differentially expressed genes associated with chromatin modification or other epigenetic regulation. The colour of the dots indicate the fold change gene expression: upregulated genes are red/orange, while downregulated genes are green. The size of the dots indicates the significance level of the pathway enrichment, -log_10_(adjusted p-value), such that larger dots have smaller p-values. “p-adj” refers to the p-value adjusted using the Benjamini-Hochberg correction for multiple testing. Only genes for which the adjusted p-value was ≤0.05 and the fold change was >2 or < -2 are shown.
(PNG)
