# Supplementary figures and images for: A transcriptomic analysis of the effects of macrophage polarization and endotoxin tolerance on the response to Salmonella
Source: PLoS One. 2022 Oct 14;17(10):e0276010. doi: 10.1371/journal.pone.0276010 (PMC9565388; doi:10.1371/journal.pone.0276010)

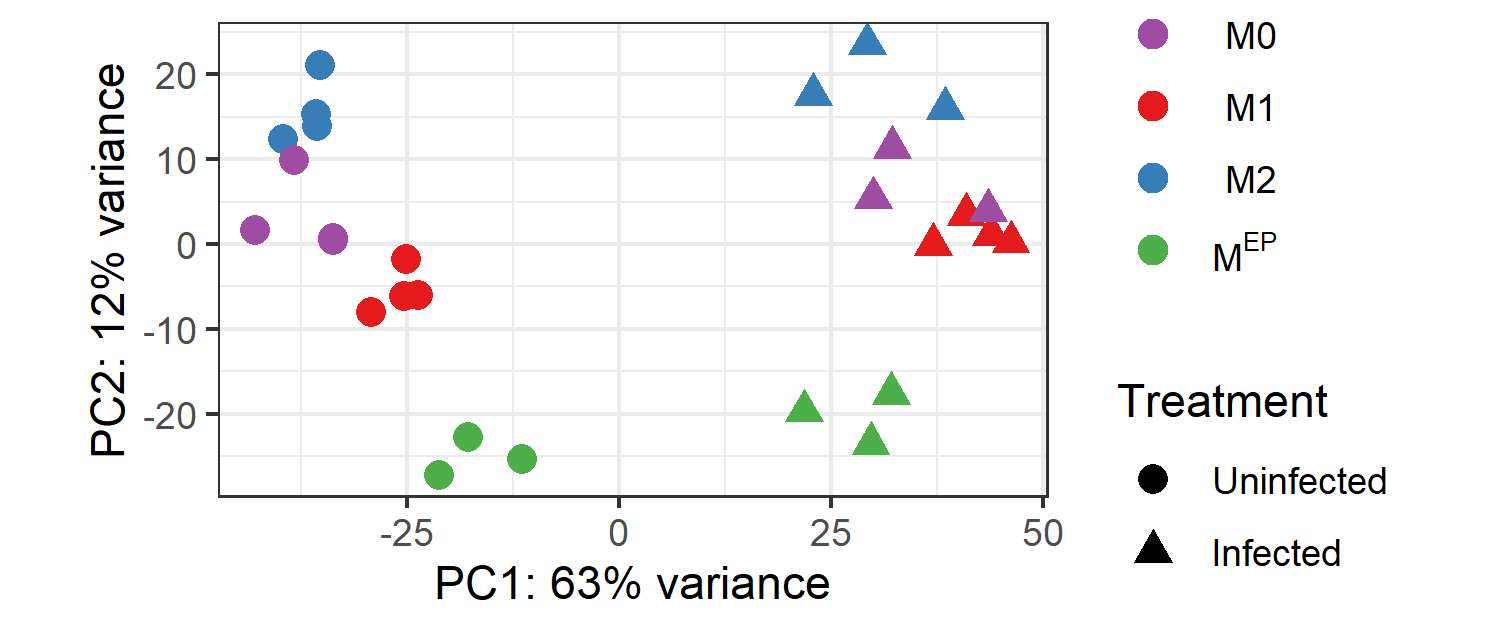

Supplement: S1 Fig — The first two principal components, plotted on the X and Y axes, summarize the greatest sources of variation between samples. (PNG) [file pone.0276010.s005.png]

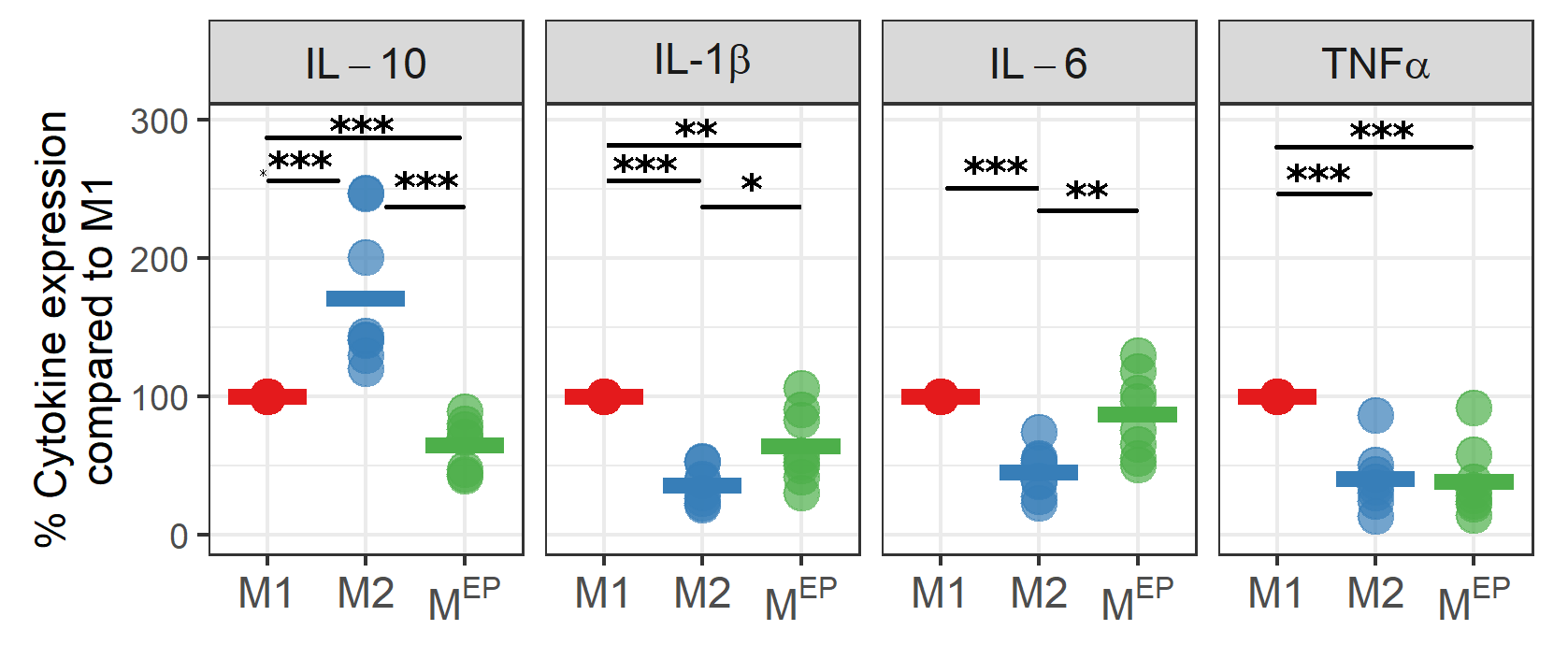

Supplement: S2 Fig — Colour corresponds to polarization, and is used for emphasis. Data is from 8 biological repeats and expressed relative to cytokine expression in M1 macrophages for the same donor; 100% represents an average 35,900pg/mL TNFα, 91,000pg/mL IL-6, 1,750pg/mL IL-10, and 345pg/mL IL-1β. Statistics were calculated by Wilcoxon test, with p-values indicated as * (< 0.05), ** (<0.01), *** (<0.001). (PNG) [file pone.0276010.s006.png]

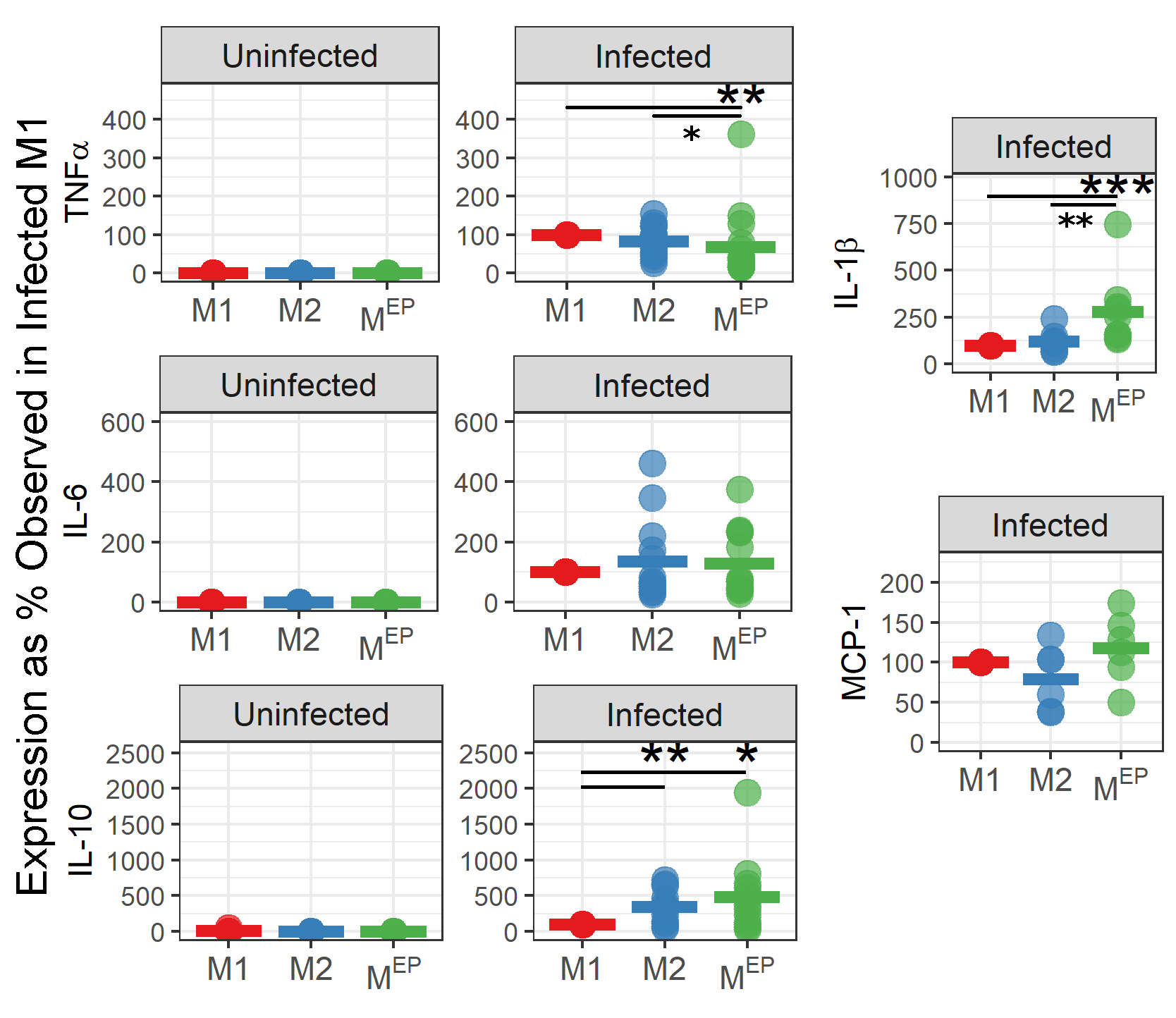

Supplement: S3 Fig — For each donor, production is expressed relative to the level observed in infected M1 macrophages; 100% represents an average 67,300pg/mL TNFα, 57,300pg/mL IL-6, 237pg/mL IL-10, 201pg/mL IL-1β, and 7,640pg/mL MCP-1. Colour corresponds to polarization, and is used for emphasis. Data represents 4 biological replicates for uninfected cells, and for infected cells 16 biological repeats for TNFα, 13 BR IL-6 and IL-10, 9 BR IL-1β, 6 BR MCP-1. Statistics were calculated by Wilcoxon test, with p-values indicated as * (< 0.05), ** (<0.01), *** (<0.001). (PNG) [file pone.0276010.s007.png]

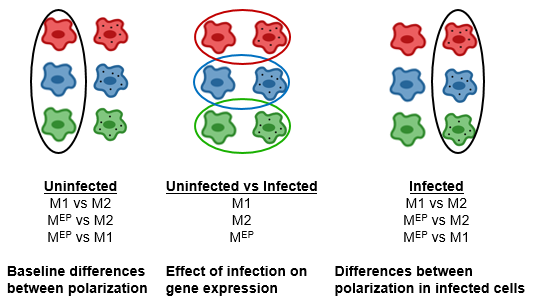

Supplement: S4 Fig — (PNG) [file pone.0276010.s008.PNG]

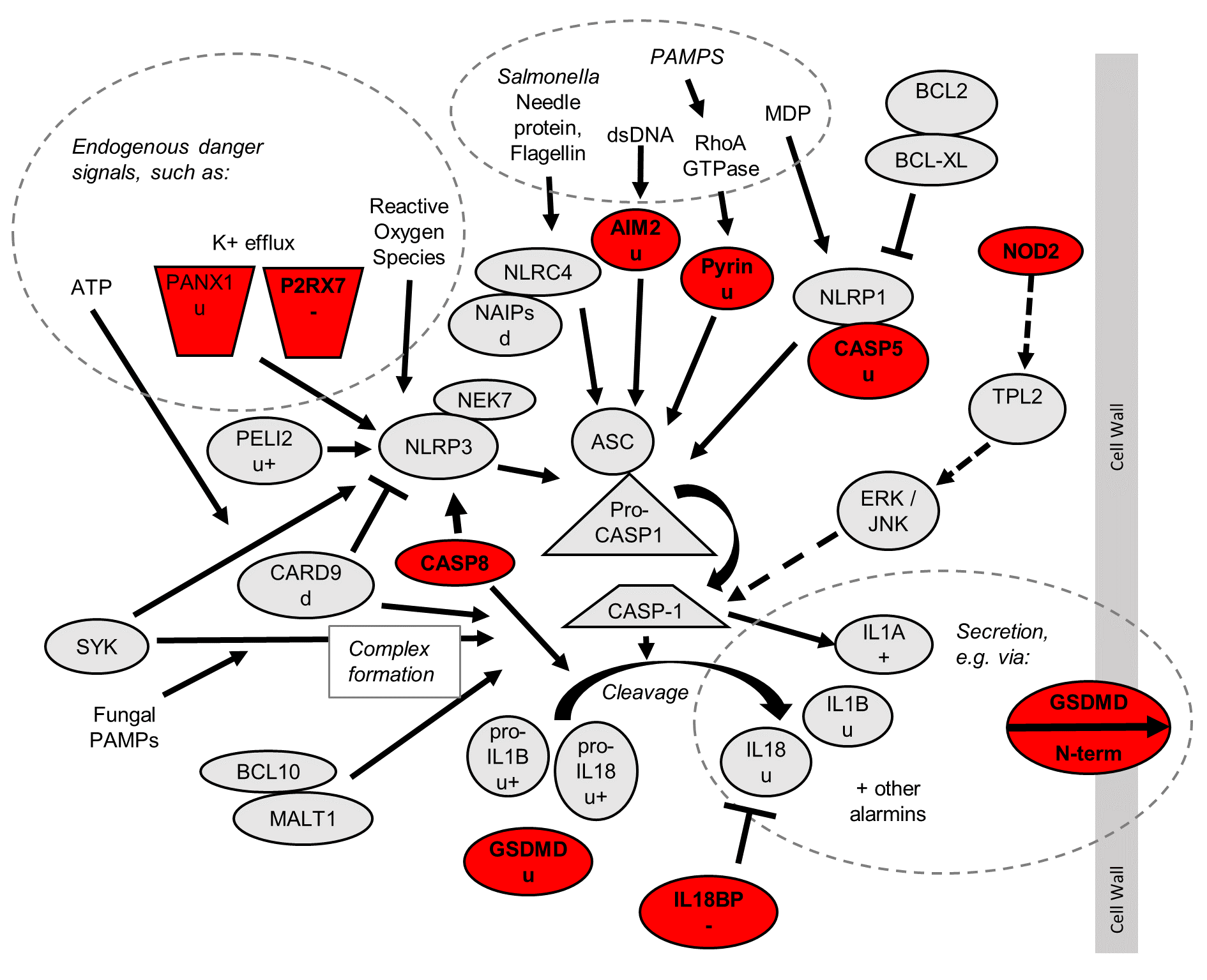

Supplement: S5 Fig — Genes upregulated in uninfected M1 vs. uninfected M2 macrophages are in red. Genes downregulated in uninfected M1 vs. uninfected M2 macrophages are in green. Genes up or downregulated in MEP vs. M2 macrophages are indicated with “u” and “d” respectively. Genes up or downregulated in MEP vs. M1 macrophages are indicated with “+” and “-” respectively. (PNG) [file pone.0276010.s009.png]

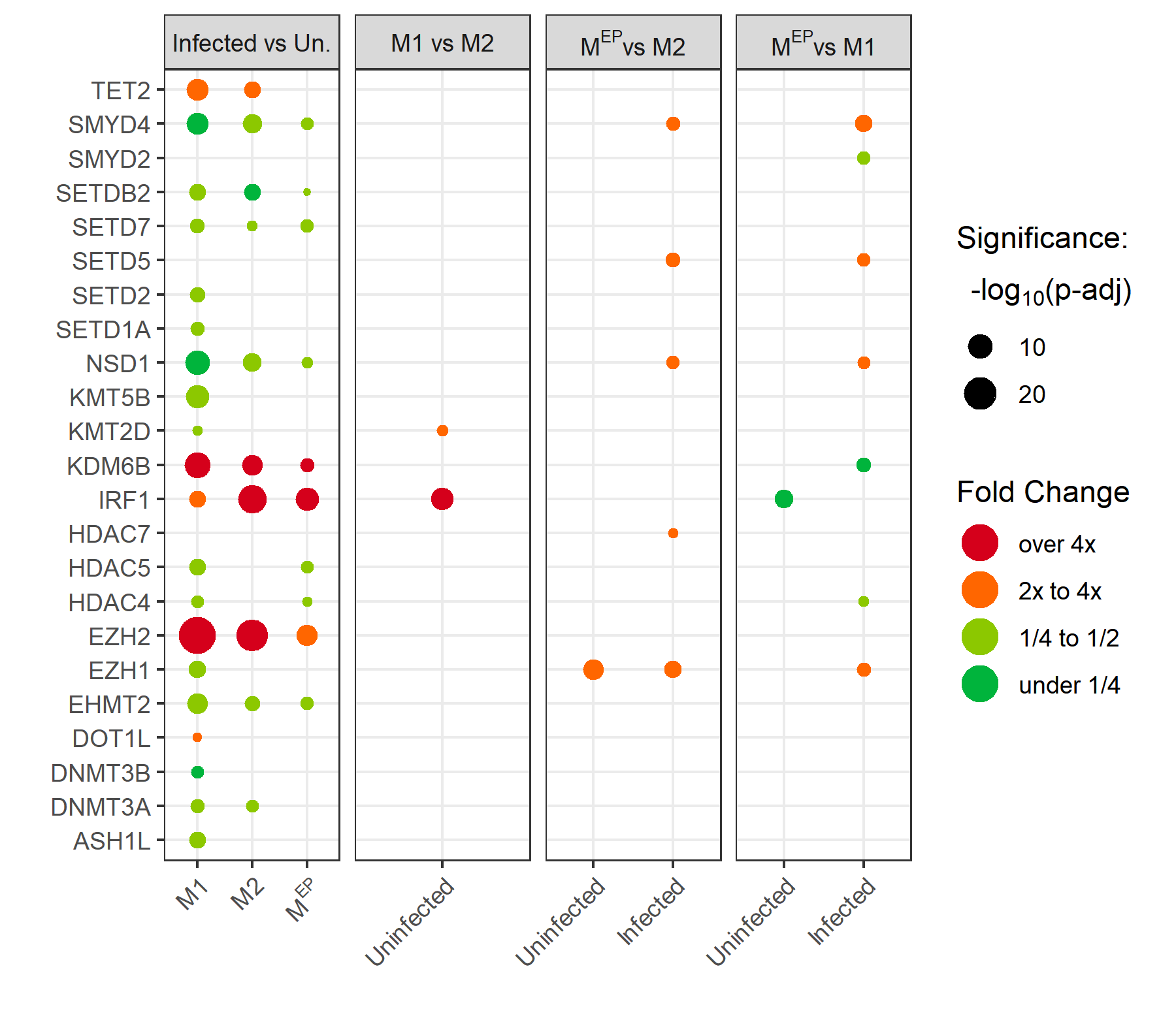

Supplement: S6 Fig — The colour of the dots indicate the fold change gene expression: upregulated genes are red/orange, while downregulated genes are green. The size of the dots indicates the significance level of the pathway enrichment, -log10(adjusted p-value), such that larger dots have smaller p-values. “p-adj” refers to the p-value adjusted using the Benjamini-Hochberg correction for multiple testing. Only genes for which the adjusted p-value was ≤0.05 and the fold change was >2 or < -2 are shown. (PNG) [file pone.0276010.s010.png]
